# Italian Translation, Adaptation, and Validation of the Novel Satisfaction Measure Assessment after Primary Total Joint Arthroplasty: The Goodman Score Questionnaire

**DOI:** 10.3390/healthcare10050769

**Published:** 2022-04-21

**Authors:** Michele Ulivi, Luca Orlandini, Valentina Meroni, Marco Viganò, Mario D’Errico, Riccardo Perrotta, Alessandra Nannini, Giuseppe M. Peretti, Laura Mangiavini

**Affiliations:** 1 IRCCS Istituto Ortopedico Galeazzi, Via Riccardo Galeazzi 4, 20161 Milano, Italy; micheleulivi@msn.com (M.U.); orlandini_luca@yahoo.com (L.O.); valedoc.meroni@gmail.com (V.M.); marioderric@gmail.com (M.D.); riccardoperrotta10@gmail.com (R.P.); alessandra.nann@gmail.com (A.N.); giuseppe.peretti@unimi.it (G.M.P.); laura.mangiavini@unimi.it (L.M.); 2 Department of Biomedical Sciences for Health, University of Milan, Via Luigi Mangiagalli 31, 20133 Milano, Italy

**Keywords:** total hip arthroplasty, total knee arthroplasty, PROMs, satisfaction, survey validation, total joint arthroplasty

## Abstract

Patient satisfaction after total joint arthroplasties (TJA) represents a key element for the evaluation of surgery success in relation to subjects’ needs and expectations. The assessment tools are applied inconsistently throughout the literature, and thus, it is difficult to compare results among different studies. Goodman et al. proposed a standardized questionnaire with strong psychometric properties for the assessment of satisfaction. The present study aims to translate, adapt, and validate the Goodman questionnaire for the Italian population. After translation and back translation, the questionnaire was administrated to 50 patients. Internal consistency, test–retest reliability, floor and ceiling effects, and construct validity were evaluated (correlation with KOOS/HOOS, SF-12 PCS/MCS, EQ-5D). Responsiveness was evaluated with respect to SF-12 PCS improvements. The Italian version of the Goodman score questionnaire demonstrated psychometric properties similar to those of the original version. The translated questionnaire showed good internal consistency (Cronbach’s alpha = 0.836) and test–retest reliability (ICC: 0.507). Moderate/strong correlations were observed between the Italian version of the Goodman score and other scores. The score significantly discriminated patients who improved from those who did not improve in SF-12 PCS after treatment. This study provides an adapted and validated Italian version of the Goodman score questionnaire, with psychometric properties similar to those of its original counterpart.

## 1. Introduction

Total hip arthroplasty (THA) and total knee arthroplasty (TKA) are cost-effective surgical procedures, performed in order to reduce pain and restore the articular function of the osteoarthritis affected joint. Every year, millions of total hip and knee replacements are performed, numbers that are expected to increase in the future due to the overall increase in mean age [1,2]. The evaluation of patient satisfaction rates and changes after surgery at different follow-ups is considered an important aspect of surgery results. In response to a steady rise in patients’ expectations, and to improve outcome measures, it is crucially important to develop new methods to evaluate these parameters in order to meet rising expectations.

Recently, in an article entitled “Assessment of a Satisfaction Measure for Use After Primary Total Joint Arthroplasty” [3], Goodman et al. proposed a questionnaire for the evaluation of patient satisfaction after THA and TKA. We proceeded to its translation into the Italian language with the aim to adapt it for the Italian patients and to assess the validity of this patient-reported satisfaction score in a different cohort of patients in terms of demography, culture, and language, as recommended by the authors in their original paper.

At our institute, all patients undergoing total joint arthroplasty are enrolled and followed through the H&K registry [4]. This Registry has also recently proved to be flexible and sensitive enough to be able to detect variations in outcome scores even in extraordinary conditions such as remote patient follow-up, which was put in place during the recent nationwide lockdown in Italy as a consequence of the COVID-19 pandemic [5].

Given the difficulty of extrapolating satisfaction from conventionally collected patient-reported outcome measures (PROMs), especially due to the weak correlation between satisfaction and pain, function, or quality of life measures, the identification of an independently measurable satisfaction evaluation system would allow for the evaluation of this crucial outcome for total joint arthroplasty (TJA) [3]. Moreover, the lack of high-quality patient satisfaction assessment tools in subjects undergoing TJA, and the extreme heterogeneity of the systems in use to measure satisfaction after prosthetic procedures, [6,7,8] suggest that the availability of cross-cultural, validated, and reliable satisfaction measurements specifically designed for this type of surgical procedure would greatly improve knowledge in the field.

For these reasons, a prospective, observational study was conducted to evaluate the validity, reliability, and effectiveness of the Italian version of the Goodman score for patient satisfaction.

## 2. Materials and Methods

### 2.1. Translation Process and Patient Enrollment

The questionnaire underwent a translation process following the internationally acknowledged method. Initially, an English-to-Italian translation of the questions was performed by two independent translators, both experts on the subject at hand and with professional English skills; afterward, the translation deemed more suitable was chosen. The questionnaires translated in Italian newly underwent a translation to English, according to the principle of back translation, made by an independent native speaking English translator, unaware of the original version. The newly obtained English version was recognized as valid following comparison with the original one. The Italian version obtained through this process (Supplementary Materials) was administered to a group of experts who inspected the final text to verify the coherence regarding 4 fundamental areas: semantic equivalence (if words had the same meaning), idiomatic equivalence (if colloquialism or idiomatic expressions were rendered comprehensible with the translation, without altering their meaning), practical equivalence (if the items in question were able to seize daily life experiences), and lastly, conceptual equivalence (if the possible conceptual differences among different cultures were thoroughly explained). After the translation process was completed, the validation process started. After signing the informed consent, the patients received the questionnaires. For the assessment of inter-rater reliability, 25 randomly chosen patients were asked to complete the questionnaire by telephone a second time, 2 weeks (±1 week) after the first administration.

### 2.2. Patients and Registry

All patients undergoing TJA at our institute are monitored from the preadmission visit through all the postsurgical time and follow-up visits planned in the registry, which systematically collects clinical data and PROMs through the submission of questionnaires and clinical evaluations, such as Knee Society Score (KSS) or Harris Hip Score (HHS) [9,10] and selected PROMs—namely, SF-12, HOOS-PS or KOOS-PS, and EQ-5D [11,12]. All patients provided informed consent before enrollment, and the study was approved by the IRB of San Raffaele Hospital in Milan (approval number CE 82/INT/2015). In addition, data on VAS and BMI were collected. These questionnaires were filled by the patient after informed consent, through different means: during preadmission or follow-up visits, from home access using the platform via a website address in order to fill the questionnaires, or through telephonic interview. Clinical scores (HHS or KSS) were determined considering both an objective clinical evaluation performed by the surgeon and the information reported by the patient during the visit. Patients included in the present study were asked to fill the Goodman score questionnaire together with other PROMs related to their participation in the H&K registry data collection.

### 2.3. Assessment Instruments

#### 2.3.1. Goodman Score

Goodman score is a questionnaire validated in 2020 in the USA [3]. It was developed to estimate patient satisfaction concerning the results obtained following TKA or THA, on a general base and regarding pain, improvement in daily life activities, domestic, gardening, and recreation. It is composed of four items, and for each item, there is a five-point Likert scale response. Each question has a score of 0–100 [3,13].

#### 2.3.2. Goodman Quality of Life

This feature was validated together with the satisfaction questionnaire; it is a question with the aim of understanding the impact of surgery on patients’ quality of life. It has just one question with a six-point Likert scale response, and the higher score reflects a worse outcome [3]. The minimal clinically important difference and the really important different thresholds correspond to the “somewhat satisfied” and “very satisfied” categories, respectively.

#### 2.3.3. KOOS/HOOS

KOOS is a multidimensional auto-evaluated questionnaire developed in the 1990s, starting from Western Ontario and McMaster Universities Osteoarthritis Index [14,15].

HOOS is a multidimensional, auto-evaluated questionnaire developed in 2003 in Sweden, starting from Western Ontario and McMaster Universities Osteoarthritis Index [16,17].

#### 2.3.4. SF-12

SF-12 questionnaire [18,19] developed in 1996 as a short alternative to SF-36 [20]. It is composed of two sections—one evaluating physical aspects (physical component summary (PCS)) and one evaluating mental fitness (mental component summary (MCS)). It was validated in 2001 and is widely used to evaluate health state and quality of life [21].

#### 2.3.5. EQ-5D

EQ-5D is a questionnaire designed to evaluate the quality of life aiming at assessing the general health status and is usually applied to a vast range of diseases/health conditions [22,23].

### 2.4. Sample Size Calculation and Statistical Analysis

The sample size was determined using the formula *n* = (10 × i), where i represents the number of items of the questionnaire [24]; the number of patients for the retest was calculated as *n* = (5 × i). Thus, 50 patients enrolled in the study and completed the questionnaire, 25 of whom participated in the retest. The subgroup of patients for retest was obtained by randomization.

The statistical analyses were performed using R software v4.0.1 (R Core Team, Vienna, Austria) and the following cited packages: Shapiro–Wilk test was used to assess the normal distribution of continuous variables, and parametric and non-parametric tests were applied according to the result of this test. Reliability was evaluated by a two-way, mixed-effects model using the “irr” package for the calculation of single measure intraclass correlation coefficient (ICC) between test and retest results. The ICC values were interpreted according to the Fleiss’ guidelines: <0.4, poor reliability; between 0.4 and 0.75, good reliability; >0.75, excellent reliability. Given the categorical nature of the QoL scale, weighted Cohen’s kappa was calculated to determine test–retest reliability. This value was interpreted as per Reiger et al. (2012) [25]. Cronbach’s alpha was calculated using the “ltm” R package for the evaluation of the internal consistency; acceptable internal consistency was assumed for a Cronbach’s alpha > 0.7. Correlations of the Goodman score with the HOOS/KOOS, SF-12 PCS, MCS, and EQ-5D were analyzed to determine the construct validity. Based on data distribution, Pearson’s or Spearmen’s r were calculated as correlation coefficients, for normal and non-normal distributed data, respectively. Correlation coefficients were considered “small” if 0.1 < r < 0.3, “medium” if 0.3 < r < 0.5, or “strong” if r > 0.5 [26]. The relative frequencies of patients scoring the worst and the best possible score were used to determine floor and ceiling effects. Effects greater than 15% were considered to be present.

## 3. Results

### 3.1. Translation, Cross-Cultural Adaptation, and Validation

No patients reported doubts about any item in the Italian version of the questionnaire. The translation from the English version and back translation demonstrated a lack of ambiguity or meaning modifications.

### 3.2. Demographics

A total of 50 patients were enrolled, 23 males and 27 females, with a mean age of 69.1 ± 10.0 years old; of those, 25 underwent total knee arthroplasty, and 25 underwent total hip arthroplasty. ASA class was 2 for 47 of them (ASA1: 2 patients; ASA3: 1 patient). Data were collected between 3 and 6 months from surgery.

### 3.3. Internal Consistency

The internal consistency of the scale based on the strength of the correlation among the 4 items was “good”, with a Cronbach’s alpha of 0.836 (CI95%: 0.724–0.904).

### 3.4. Reliability

In total, 25 patients completed the retest (14 men and 11 women; mean age, 71.6 ± 8.7 years old; 11 THA, 14 TKA). In this subset of patients, the mean satisfaction score at test was 80 ± 17.7, and at retest, the score was 86 ± 13.7. Interclass correlation coefficient (ICC) between test and retest assessment was 0.507, showing good reliability (CI95%: 0.162–0.745, *p* = 0.003). Cohen’s kappa was calculated for QoL scale, resulting in good reliability (0.508, *p* < 0.001). For comparison, in this cohort, HOOS/KOOS showed excellent reliability, with ICC of 0.867 (*p* < 0.001), while EQ-5D (0.595, *p* < 0.001), SF12 PCS (0.588, *p* < 0.001) and MCS (0.410, *p* = 0.020) showed good reliability.

### 3.5. Feasibility

All patients completed the questionnaires, with no missing responses. The ceiling effect, calculated as the percentage of patients scoring 100, was 30.0% (*n* = 15) as regards the satisfaction score. For comparison, HOOS/KOOS, SF12-PCS, and MCS in the same cohort demonstrated a ceiling effect of 2%, 0%, and 0%, respectively. Nevertheless, EQ-5D showed a similar ceiling effect, to that of the satisfaction score (28%). The floor effect was 0 for satisfaction, indicating that no patients scored 0 for all items. The same floor effect was observed for all the other scales.

### 3.6. Construct Validity

The correlation of the satisfaction questionnaire was moderate or strong and significant with KOOS/HOOS total scores and subscales, as well as with EQ-5D, EQ-5D VAS, and PCS subscale of SF-12. A weak and non-significant correlation was observed with MCS. Similarly, the quality-of-life subscale of the satisfaction questionnaire also demonstrated a moderate/strong correlation with all the other scores (Table 1), except MCS, which demonstrated a weak significant correlation.

### 3.7. Responsiveness

**Figure 1:**
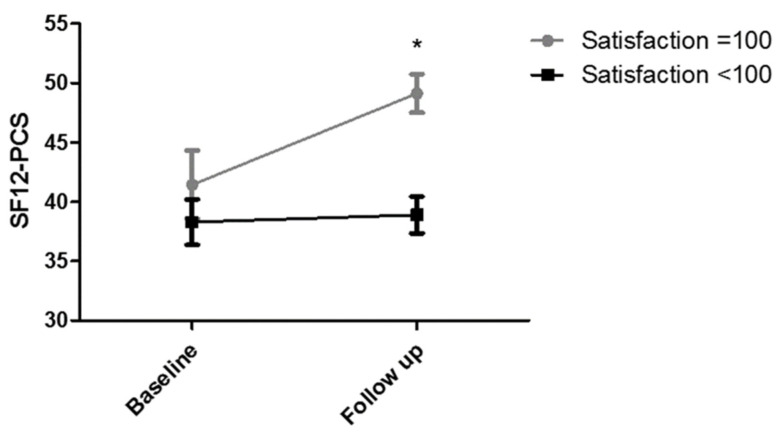
SF12-PCS improvement in patients scoring 100 (maximum) in the satisfaction questionnaire and in patients scoring less than 100. * *p* < 0.05 between groups.

Satisfaction questionnaire was able to discriminate patients with greater improvements, measured by SF12-PCS. In fact, patients scoring 100 in the satisfaction questionnaire had a higher improvement, compared with lesser-satisfied patients, with mean increment of 7.7 ± 10.4 and 0.6 ± 11.8 points of PCS (*p* = 0.047), respectively (Figure 1). This difference was not observed for MCS (satisfaction = 100: 7.8 ± 10.1; satisfaction < 100; 9.4 ± 14.7; *p* = 0.665).

## 4. Discussion

Many studies related to “patient satisfaction” were performed over time, and a definition is derived from Ware et al. in 1973, who believed satisfaction to be connected to the patient’s expectations and also to pain relief and functional improvement [27]. Other Authors suggest otherwise, indicating that many factors, both internal and external, contribute to determining patient satisfaction [28,29]. An evaluation of the available literature reports shows how difficult it is to find a specific and clearly defined measure to determine patient satisfaction. This is even more apparent when searching for a specific score validated in the Italian population who underwent total joint replacement. Usually, to obtain an indirect measure of patient satisfaction rate, it is necessary to rely on different PROMs such as HOOS-PS [11] or KOOS-PS [12], SF-12 [18,19,21], Oxford Hip and Knee Score (OHS and OKS), and Western Ontario and McMaster University (WOMAC) Osteoarthritis Index [30,31]. Each of these measurements evaluates many different aspects such as pain, mobility, and physical and mental states, which are known to significantly affect individual satisfaction [29]. Satisfaction has been discussed in systematic reviews, concluding that studies evaluating this aspect suffered from extreme heterogeneity and from the lack of a standardized method to measure it [6]. In addition, questionnaires focused on mental health and status, such as SF-12 MCS, demonstrated thus far low effectiveness in capturing improvements in the subjects who underwent joint arthroplasties [32].

The questionnaire for the assessment of satisfaction proposed by Goodman et al. [3] was developed specifically for patients who underwent TJA, thus introducing a specific and standardized tool for measuring postsurgical satisfaction in these types of patients.

The results of our study, besides highlighting the relative easiness and factual consistency of the translation into the Italian language, allow the successful validation of the Italian adaptation of the questionnaire, which demonstrated analogous properties, compared with the original English questionnaire [3]. Indeed, compared with the original questionnaire, the Italian version showed similar internal consistency (0.836 vs. 0.88), ICC between test and retest (0.51 vs. 0.51–0.61), and correlations with the other scores (KOOS/HOOS, SF-12 PCS, EQ5D) with exception of SF-12 MCS. The same could be said in relation to the QoL scale. A ceiling effect was observed in our cohort, and it was in accordance with the strong ceiling effect described by the original authors. Nevertheless, in the short term (3–6 months) following TKA and THA surgeries, improvements tend to be extremely positive [33,34], especially when performed in high-volume specialized centers such as the one where this study was conducted [35], and therefore, such an effect on satisfaction was expected.

Recently, a Spanish adaptation and validation of the Goodman score questionnaire were published, reporting higher Cronbach’s alpha (0.95) and test–retest ICC (0.87) than those of both the Italian and English versions [36]. Besides cultural and language differences, the higher indices of the Spanish adaptation may be due to the inclusion in the study of patients undergoing THA only, as well as the use of a larger cohort than the one described in the present study. Indeed, the original article showed non-negligible differences in the psychometric properties of the questionnaire in the assessment of patients undergoing TKA or THA [3].

Compared with the original validation study by Goodman et al., this study administered the questionnaire to a markedly different cohort of patients in terms of older age, language, and cultural background; therefore, it further validated and confirmed the adoptability of this satisfaction questionnaire in patients undergoing primary total knee and hip replacement in Italian speaking areas.

Limitations of our study are the short-term follow-up assessment period after surgery and the small number of patients included in the study according to the sample size calculation, especially considering the inclusion of two different types of surgeries. However, since the validated questionnaire will be included in the routine activity of the institute’s registry of hip and knee replacement, data about a larger cohort of patients will be available soon, and the results in the medium and long term (5–10 years) will be monitored for all patients, including those enrolled in the present study. This will allow for further evaluation of the questionnaire.

## 5. Conclusions

Patient satisfaction is of paramount importance since it underlies the clinical success of the procedures, but also measures the effectiveness of these therapies in matching the patients’ specific needs and expectations. Therefore, the validation of solid and consistent questionnaires in different languages and cultural contexts will allow for the systematic assessment of this aspect and provide the basis for the scientific community to compare the results in a consistent way. This study successfully provides an adapted and validated Italian version of the Goodman score questionnaire.

## Figures and Tables

**Table 1:** Correlation between Goodman score and Goodman QoL and validated PROMs.

Scale	Correlation with Goodman Score ^1^	Correlation with Goodman QoL ^1^
HOOS/KOOS Pain	0.664 (*p* < 0.001)	−0.507 (*p* < 0.001)
HOOS/KOOS Symptoms	0.509 (*p* < 0.001)	−0.627 (*p* < 0.001)
HOOS/KOOS ADL	0.586 (*p* < 0.001)	−0.634 (*p* < 0.001)
HOOS/KOOS Sport	0.579 (*p* < 0.001)	−0.499 (*p* < 0.001)
HOOS/KOOS QoL	0.460 (*p* = 0.001)	−0.489 (*p* < 0.001)
Total HOOS/KOOS	0.677 (*p* < 0.001)	−0.655 (*p* < 0.001)
SF-12 MCS	0.254 (*p* = 0.075)	−0.313 (*p* = 0.027)
SF-12 PCS	0.552 (*p* < 0.001)	−0.444 (*p* = 0.001)
EQ-5D	0.648 (*p* < 0.001)	−0.496 (*p* < 0.001)
EQ-5D VAS	0.4373 (*p* = 0.001)	−0.426 (*p* = 0.002)

^1^ Data are reported as Spearman correlation coefficient and *p* value of the correlation test within brackets.

## Data Availability

The data presented in this study are openly available in the OSF repository at https://osf.io/7nzw4/?view_only=297c7c6173af443f896473c2af5125ab (accessed on 19 April 2022).

## Supplementary Materials

The following supporting information can be downloaded at: https://www.mdpi.com/article/10.3390/healthcare10050769/s1, Goodman Score–Italian Version.
