# Supplementary material for: Italian Translation, Adaptation, and Validation of the Novel Satisfaction Measure Assessment after Primary Total Joint Arthroplasty: The Goodman Score Questionnaire
Source: Healthcare (Basel). 2022 Apr 21;10(5):769. doi: 10.3390/healthcare10050769 (PMC9141051; doi:10.3390/healthcare10050769)
Supplement: Supplementary file 1 [file healthcare-1668629-supplementary.pdf › healthcare-1668629-supplementary.pdf]

Goodman Score Tradotto e validato in italiano

Domande sul grado di soddisfazione:

1) **Qual è il Suo grado di soddisfazione in relazione ai risultati dell'intervento al ginocchio/anca nelle aree seguenti?**
   (Si prega di cerchiare una risposta per ogni domanda).

   In caso di operazione a entrambi gli arti, risponda pensando alla Sua situazione generale.

	Molto Soddisfatto	Abbastanza soddisfatto	Né soddisfatto né insoddisfatto	Abbastanza Insoddisfatto	Molto insoddisfatto
a. In termini di sollievo dal dolore?	O	O	O	O	O
b. In termini di miglioramento della capacità di svolgere faccende domestiche o attività di giardinaggio?	O	O	O	O	O
c. In termini di miglioramento della capacità di svolgere attività ricreative?	O	O	O	O	O
d. Nel complesso, qual è il suo grado di soddisfazione in relazione ai risultati dell'intervento al ginocchio/anca?	O	O	O	O	O

2) Domanda sulla qualità della vita:

In che misura l'intervento al ginocchio/anca ha migliorato la Sua qualità della vita?

Un miglioramento superiore alle aspettative	Un ottimo miglioramento	Un moderato miglioramento	Un lieve miglioramento	Nessun miglioramento	La mia qualità della vita è peggiorata
O	O	O	O	O	O
